# Composite Thiophene-Based Nanoparticles: Revisiting the PEDOT:PSS/P3HT Interface for Living-Cell Optical Modulation

**DOI:** 10.1021/acsami.5c02115

**Published:** 2025-04-04

**Authors:** Gabriele Tullii, Christian Bellacanzone, Hansel Comas Rojas, Francesco Fumagalli, Carlotta Ronchi, Anthea Villano, Federico Gobbo, Marco Bogar, Barbara Sartori, Paola Sassi, Giulia Zampini, Giulia Quaglia, Loredana Latterini, Heinz Amenitsch, Maria Rosa Antognazza

**Affiliations:** † Center for Nano Science and Technology, Istituto Italiano di Tecnologia, Via Rubattino 81, 20134 Milano, Italy; ‡ European Commission, Joint Research Centre (JRC), Ispra, Italy; § Physics Dept., Politecnico di Milano, P.zza L. da Vinci 32, 20133 Milano, Italy; ∥ Department of Engineering and Architecture, University of Trieste, Via Alfonso Valerio 6/1, 34127 Trieste, Italy; ⊥ Institute of Inorganic Chemistry, Graz University of Technology, Stremayrgasse 9/4, A-8010 Graz, Austria; # Dipartimento di Chimica, Biologia e Biotecnologie, Università di Perugia, Via Elce di Sotto, 8, 06123 Perugia, Italy

**Keywords:** organic bioelectronics, PEDOT:PSS, endothelial cells, P3HT, nanoparticles, light

## Abstract

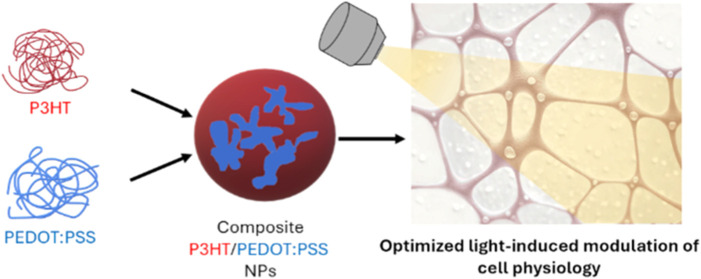

Organic semiconducting nanoparticles (NPs) have been attracting increasing attention for their diverse applications in biotechnology, especially as photoactive materials for spatially controlled optical modulation of living-cell functions. Different approaches to optimize their efficacy and reliability have been recently attempted, including control of photophysical/-chemical properties, ad hoc tailoring of materials synthesis, and functionalization with biological moieties. Another promising strategy is offered by the realization of composite light-sensitive NPs, with a supramolecular architecture. This work reports on the fabrication and characterization of polymer NPs based on poly(3-hexylthiophene-2,5-diyl) (P3HT) and poly(3,4-ethylenedioxythiophene):poly(styrenesulfonate) (PEDOT:PSS) as prototypical examples of fully biocompatible, semiconducting and conducting materials, respectively. This peculiar NP architecture, with conducting islets distributed within the semiconducting phase, translates into optimization of charge dissociation and electron-transfer efficiency, as well as photocurrent generation increase by about an order of magnitude. As an example of relevant physiological interest, effective optical modulation of angiogenesis, driven by NPs, is demonstrated in primary human endothelial cells. The reported strategy is of general validity and broadens the tools available for spatiotemporally controlled, optical modulation of living-cell functions *via* engineering of the NP architecture and processes at the interface with living cells.

## Introduction

Over the last few decades, semiconducting polymers have emerged as ideal candidates for optical modulation of cellular physiology, acting as biocompatible, soft, and conformable phototransducers.^1−4^ Light in conjunction with semiconducting polymers allows the biological functions to be triggered with excellent spatial (submicrometer) and temporal (∼1 ms) resolution, in a wireless manner, without the need for viral transfection. These materials have been exploited for optical modulation of the physiological activity of several biological models *in vitro*, including primary neurons, excised retinal tissues, stem cells, and brain slices.^5−8^*In vivo*, they have been employed for a number of promising applications, including the expression of opsin proteins in hydra animal models and artificial visual prosthesis.^3,9^ In view of *in vivo* applications, semiconducting polymers in the form of nanoparticles (NPs) are particularly interesting, given their higher translational potential: in fact, they can be administered by several, minimally invasive routes, and they can be functionalized in order to target specific sites, down to the subcellular length scale.^10^ Among other interesting application opportunities, we have recently demonstrated that conjugated polymer NPs coupled with visible-light excitation efficiently control the angiogenic process, *i.e.*, the formation of new blood vessels.^11,12^ Depending on the stimulation parameters, *i.e.*, power density of the light stimulus, type of material, and morphology (thin film, nanoparticles), it is possible to achieve either process enhancement (promising for therapeutic angiogenesis applications) or inhibition (promising for treatment of hypervascular conditions, like in tumors). Interestingly, we showed that there is a strong link between the angiogenesis modulation and light-triggered reactive oxygen species (ROS) production at the conjugated polymer/water interface. However, for full exploitation of *in vivo* therapy, further optimization of the photoelectrochemical efficiency is needed to boost the phototransduction efficiency while minimizing the required light power density. A possible approach is the increase of the interfacial area between the photoactive polymer and the electrolyte, where oxygen reduction processes take place, as recently reported in porous polymer nanoparticles.^13^ However, full control of the NP morphology and supramolecular organization is in this case hardly achievable, the repeatability/scalability of the fabrication process is limited, with a critical impact on the creation of the diffused interface between the conjugated polymer and the cell cytosol, and in any case, the overall photoelectrochemical efficiency is limited by the presence of defects/charge traps at the interface between the semiconducting polymer and the electrolyte. Thus, alternative approaches are required to produce biocompatible polymer NPs, with high photoelectrochemical efficiency at low-light density, compatible with *in vivo* applications.

Herein, we report an effective strategy based on the introduction, within the NP bulk, of a buried interface that is able to maximize charge dissociation. To achieve this goal, we developed composite NPs composed of a conjugated-polymer-based semiconducting shell and a conducting/semiconducting phase, acting as a dissociation interface. Poly(3-hexylthiophene-2,5-diyl) (P3HT) was selected as the photoactive material, given its consolidated role as a phototransducer for the ROS-guided modulation of cell functions.^11,13−16^ Poly(3,4-ethylenedioxythiophene):poly(styrenesulfonate) (PEDOT:PSS) served as the charge-dissociation phase, which given its hole-transporting/electron-blocking properties is extensively employed in the bioelectronics field.^17,18^ We performed in-depth characterization of optical, physicochemical, and structural properties of the composite NPs, and we explored the influence of the PEDOT:PSS charge-separation phase on photocurrent generation. Most importantly, we demonstrate that the presence of the PEDOT:PSS phase increases the light-induced antiangiogenic efficacy of the P3HT-based NPs (up to 36% light *vs* dark angiogenesis inhibition).

Since the approach presented here is of general validity, our results open the way for further optimization of NPs suitable for *in vivo* use in all those therapeutic applications that require minimally invasive, remotely controlled modulation of intracellular redox balance and ROS generation under eustress and distress conditions.^19^

## Results and Discussion

### Synthesis and Characterization of PEDOT:PSS/P3HT NPs

Composite NPs, where the P3HT p-type semiconducting material is mixed with an n-type material, such as [6,6]-phenyl-C61-butyric acid methyl ester (PCBM), have been intensively studied and characterized in the last two decades.^20^ The p–n architecture significantly increases (up to several orders of magnitude) the long-lived charge photogeneration quantum yield of P3HT, amounting at 10^–3^ to 10^–4^ in the pristine material.^21^ This effect is due to the increased surface area available for charge separation, and it has been widely employed to enhance the efficiency of organic photovoltaics. A few reports demonstrated the possibility of employing P3HT-based NPs bearing a p–n structure in biophotonic applications for optical stimulation of living-cell activity.^22,23^ Quite surprisingly, and to the best of our knowledge, NPs composed of P3HT and PEDOT:PSS have not been reported so far. Nevertheless, the selection of P3HT and PEDOT:PSS is strategic for live-cell photomodulation for several reasons: (i) P3HT and PEDOT are both biocompatible, and both have been reported for applications in biology and physiology, also in chronic settings;^3,13,13,16,17,24^ (ii) PEDOT:PSS shows excellent electronic and ionic conductive properties in a biological environment;^17^ (iii) P3HT maintains its semiconducting properties in a biological environment, and it is widely accepted as an ideal transduction material for optical modulation of living cells and tissues.^8,25−27^

Thus, the implementation of composite injectable particles encompassing both materials looks like a logical choice, though their realization and optimization are not straightforward.

**Figure 1:**
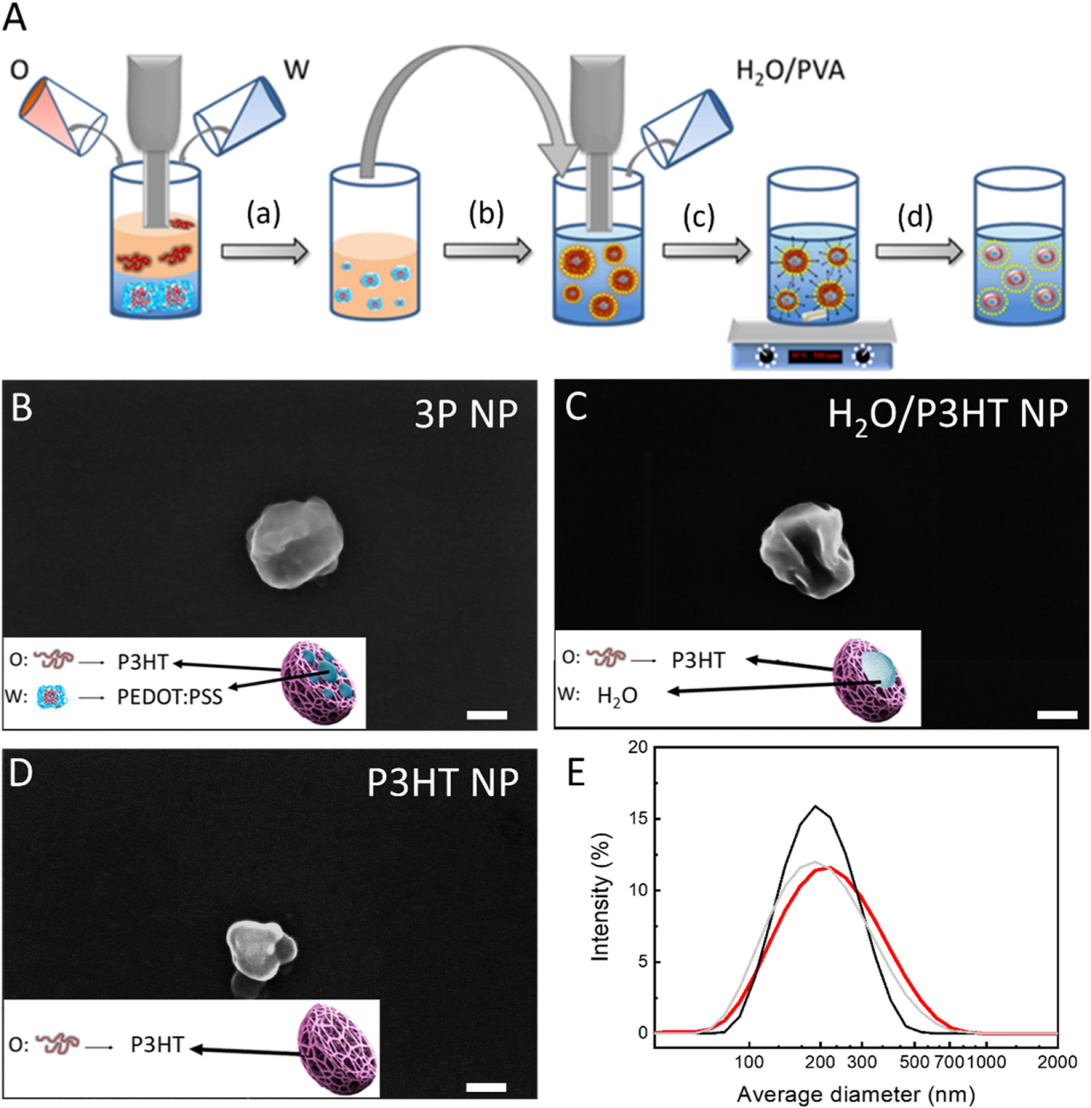
(A) Schematic drawing of the fabrication steps of the composite NPs. Representative SEM images of 3P (B), H_2_O/P3HT (C), and P3HT NPs (D). Scale bars: 100 nm. (E) Size distribution of NP dispersion, reported in logarithmic scale, as obtained by DLS (3P, H_2_O/P3HT, and P3HT, shown as red, gray, and black lines, respectively).

As a first step, we fabricated NP dispersions and characterized their structural organization. PEDOT:PSS/P3HT nanoparticles (3P NPs) were realized by employing the double mini-emulsion method (Figure 1A). Briefly, a first water (w)-in-oil (o) emulsion was obtained (step a, Figure 1A) by mixing the water-based PEDOT:PSS dispersion (w) with a chloroform solution of P3HT (o). The as-obtained first emulsion was ultrasonically treated and then added to a water dispersion of poly(vinyl alcohol) (PVA), a stabilizing agent (step b, Figure 1A). A subsequent irradiation with ultrasound induced the formation of a water-in-oil-in-water double emulsion (step c, Figure 1A). After complete evaporation of the organic solvent (step d, Figure 1A), 3P NPs were formed. The NP morphology was investigated by scanning electron microscopy (SEM). A representative SEM image of a single 3P NP is shown in Figure 1B. Two types of control dispersions lacking the PEDOT:PSS component were also considered: (i) H_2_O/P3HT NPs, in which we followed the same fabrication steps as in the 3P NPs case, but we substituted PEDOT:PSS with deionized water (Figure 1C); (ii) P3HT NPs, fabricated from a single emulsion, obtained by mixing the P3HT organic solution directly with PVA in water and by following steps c and d (Figure 1D).

Average hydrodynamic diameters of the NPs have been measured by dynamic light scattering (Figure 1E), obtaining comparable values for 3P, H_2_O/P3HT and P3HT NPs: 203.4 ± 4.2 nm (polydispersity index, PDI = 0.223), 187.7 ± 3.2 nm (PDI = 0.196), and 185.5 ± 1.7 nm (PDI = 0.097), respectively.

**Figure 2:**
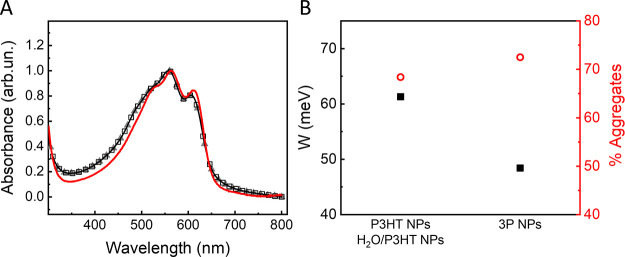
(A) Normalized absorption spectra of 3P NPs (red line), H_2_O/P3HT NPs (gray line, filled triangles), and P3HT NPs (black line, empty squares). (B) Free-exciton bandwidth (W, left; filled black squares) and relative fraction of aggregates (right, empty circles) calculated for 3P NPs and control P3HT NPs and H_2_O/P3HT NPs.

Normalized ultraviolet–visible (UV–vis) absorption spectra of the three NP dispersions are depicted in Figure 2. Absorption features typical of P3HT NP dispersions,^28^ around 525, 567, and 621 nm, are present in all cases, and they are assigned to the 0–2, 0–1, and 0–0 vibronic transitions, respectively.^29,30^ Interestingly, H_2_O/P3HT and P3HT NPs spectra are closely overlapped; the 3P NP spectrum instead presents a bathochromic shift as well as an increase in the relative intensity of the 0–0 vibronic peak. Both these features possibly indicate a higher crystallinity of the P3HT polymer.^31^ Extensive literature reports show that crystalline-grade P3HT-based NPs, directly proportional to the charge mobility, may depend on many factors, including the surfactant type, the fabrication method, the organic solvent employed to disperse the polymer, as well as the presence of other components blended with P3HT, like fullerenes.^32−34^ In the present case, the very same fabrication parameters and materials were employed for all tested NPs; thus, we hypothesize that the crystallinity increase is due to the presence of PEDOT:PSS within the 3P NP architecture.

The fraction of aggregates within the NP suspensions can be roughly estimated by decomposing the absorption spectra into semicrystalline and amorphous fractions and by subtracting the spectrum of amorphous P3HT in chloroform solution.^30^ The fraction of aggregates within the NP suspensions is slightly higher in 3P NPs (72.5%) than in H_2_O/P3HT or P3HT NPs (68.4% in both cases) (Figure 2B, right axis). In more detail, information about the characteristics of the aggregated structure can be retrieved by comparison of the relative vibrational peak intensities, according to the H/J aggregate model developed by Yamagata and Spano.^35,36^ The intensity ratio between the peaks associated with the origin (0–0) and the first vibronic satellite (0–1) transitions (A_0–0_/A_0–1_), in fact, allows us to obtain information on (i) the dominant coupling type, *i.e.*, H-type (face-to-face, A_0–0_/A_0–1_ <1) or J-type (end-to-end, A_0–0_/A_0–1_ >1) aggregates and (ii) the coupling magnitude (exciton bandwidth, W).^37,38^ J-aggregates usually prevail in systems with a high degree of planarity of thiophene rings; conversely, weakly coupled H-type aggregates are typical of more pronounced interchain coupling and intrachain torsional disorder. In the present case, the ratio A_0–0_/A_0–1_ is <1 for all NP dispersions, thus suggesting the major presence of H-aggregates. However, 3P NPs are characterized by a slightly higher value as compared to the control cases (Table 1). Free-exciton bandwidth values (*W*, Figure 2B, left axis) were calculated by employing the following expression
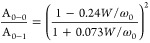
1where ω_0_ is the vibrational energy of the C=C symmetric stretch that dominates the coupling to the electronic transition (assumed to be 0.18 eV).^38,39^ The value obtained for 3P NPs is sizably lower than in the case of both control NPs (48.4 *vs* 61.3 meV), thus indicating an increased conjugation length and chain ordering.^38^ Overall, data reported in Figure 2 and Table 1, *i.e.*, the increase of the A_0–0_/A_0–1_ ratio, along with the reduction of *W*, observed in 3P NPs point out that the presence of PEDOT:PSS determines the formation of more ordered P3HT aggregates and a longer conjugation length.

**Table 1:** Central Absorption Wavelength and Intensity Ratio (A_0—0_/A_0—1_) Associated with the Vibronic Transition Recorded in 3P NPs and Relevant P3HT NP Control Samples

NPs	λ_0–2_ [nm]	λ_0–1_ [nm]	λ_0–0_ [nm]	A_0–0_/A_0–1_
3P	531	564	612	0.85
H_2_O/P3HT	521	557	606	0.81
P3HT	521	557	606	0.81

The impact of the PEDOT:PSS component on spectroscopic features of 3P NPs was further confirmed by micro-Raman measurements (Figure S1). In order to isolate the sole PEDOT:PSS contribution to the Raman spectrum, we show the signal difference between the 3P NPs and control H_2_O/P3HT NPs. The comparison with the bare PEDOT:PSS NPs Raman spectrum confirms the presence of PEDOT:PSS component within the 3P NP architecture, since evidences that the Raman peaks in the frequency range between 1420 and 1450 cm^–1^, assigned to the C_α_=C_β_ symmetric stretch of the constituent five-membered ring of PEDOT, are evidenced in both curves.^40^ Interestingly, one should also notice a sizable spectral shift of the C_α_=C_β_ feature from 1420 cm^–1^ in PEDOT:PSS particle dispersion to 1436 cm^–1^ in the 3P NP spectrum. In addition, the ratio between the C–C inter-ring stretching (1260 cm^–1^) and the C=C symmetric stretch is lower in 3P NPs, as compared to control PEDOT:PSS samples. The shift results from a change from the quinoid structure to the benzoid structure in the PEDOT chains, possibly associated with a conformational change in the PEDOT:PSS chains from a predominantly linear structure to a more mixed linear-coil formation. Interestingly, in the present work, such a change possibly accounts for the interaction between P3HT and PEDOT:PSS, determining conformational changes, and it may even facilitate a stronger interaction between the chains, as already suggested by Ouyang et al.^41,42^

**Figure 3:**
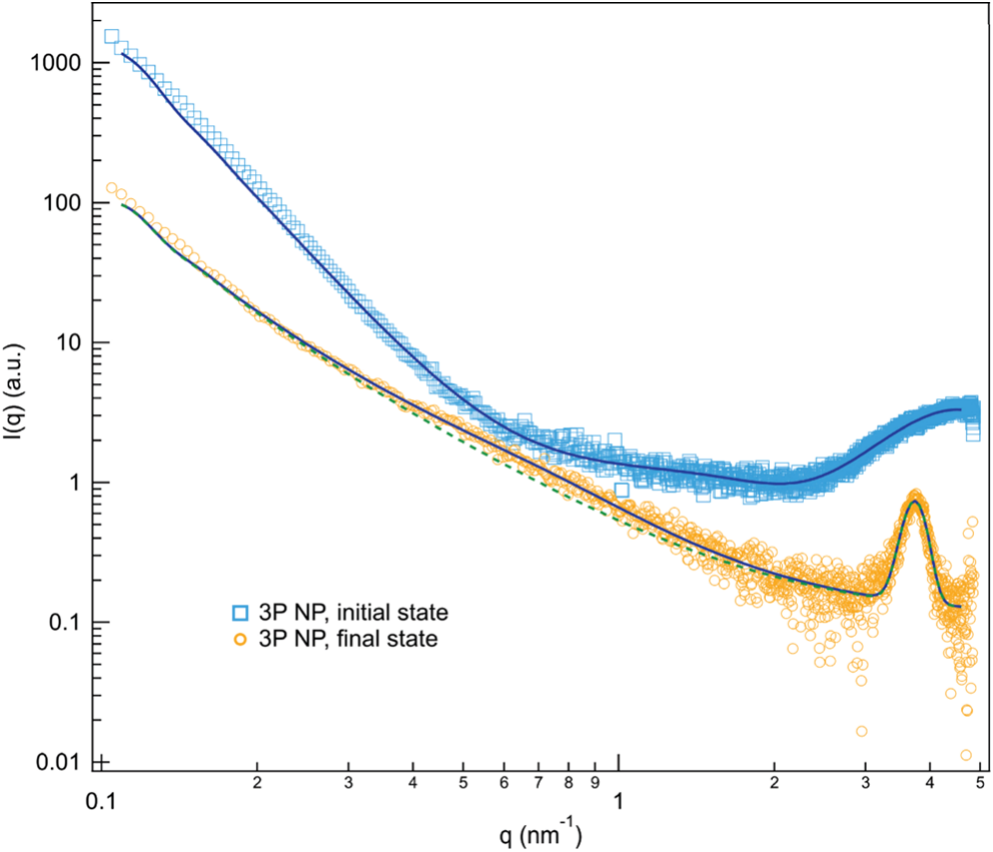
Scattering patterns representative of the formation of 3P NPs, before (initial state, time point (c), Figure 1A) and after (final state, time point (d)) evaporation of the organic solvent. Solid lines represent the best fits to the scattering data according to the model including the Teubner–Stray (TS) contribution. The dotted line shows the fitting output on the data collected from the final status without the TS contribution, *i.e.*, by hypothesizing a core–shell NP architecture.

Structural organization of PEDOT:PSS and P3HT components within the 3P NP architecture is of critical importance for possible applications in nanomedicine since it governs all of the interfacial processes at play in the modulation of cell physiological activity. In particular, the morphological organization of the electron-donor and electron-acceptor components is expected to critically determine the efficiency of electron-transfer reactions at the donor/acceptor interface, as well as at the polymer/electrolyte interphase, thus impacting the overall interest of the NP architecture engineering and optimization. Structural organization of 3P NPs has been studied by small-angle X-ray scattering (SAXS) measurements (Figure 3). The scattering patterns recorded before and after evaporation of the organic solvent from the solution (time points (c) and (d) of Figure 1A, respectively) are compared.

When solvent evaporation starts, the dispersed PEDOT:PSS NPs are responsible for generating the feature in the low-q region. Afterward, the crystallization of P3HT forming the shell of the NPs is underlined by the appearance of a diffraction peak in the high-q regime. From data fitting, the peak was found centered at 3.76 nm^–1^, corresponding to the (100) reflection of the lamellar packing of crystalline P3HT.^33^ In addition, in the final state, the scattering pattern is characterized by a bump rising around 0.5 nm^–1^ and by a rising slope at low-q values. A core–shell NP organization was initially hypothesized, but this model was not sufficient to describe all of the features of the scattering curves (Figure 3, dotted line). Thus, a different fitting approach was necessary, and we considered the formation of multicore PEDOT:PSS islets within P3HT, modeled by introducing a Teubner–Stray-like contribution in the fitting.^43^ Data are nicely reproduced; moreover, from the fitting output, it was found that PEDOT:PSS domains are characterized by a correlation length of about 1.8 nm and are at 11.5 nm distance, on average.

The presence of PEDOT:PSS multicores within the 3P architecture was further confirmed by X-ray photoelectron spectroscopy (XPS) measurements. Due to their strong interaction with matter, X-ray photoelectrons exhibit a characteristic attenuation length within a solid of only a few nanometers, depending on the photon energy and material characteristics. Therefore, the depth from which information is obtained in a typical XPS experiments (3 times the attenuation length or ca. ≈ 95% of the observed photoelectron signal) is limited for polymers to ca. 11 nm.^44^ For this reason, the use of the surfactant (PVA, see the Materials and Methods section) in the NP fabrication protocol must be taken into account. Atomic concentrations derived from survey spectra describing the surface chemistry characteristics of 3P NPs and control systems (P3HT NPs and PEDOT:PSS) are shown in Table S1 and Figure S2. As expected, the surfaces of all samples are composed mainly of C, O, and, to a lesser extent, S (see survey spectra in Figure S2). Traces of Na, Cl, and Si are also detected as contaminants coming from the dispersion solution and from sample manipulation. The atomic concentration ratios of O/C and S/C are reported in Table S1, together with the stoichiometric reference values. Superimposed to a certain amount of adventitious carbon interference, comparison of measured and reference values for the P3HT NP system indicates a substantial contribution of the PVA surfactant coating to the nanoparticles’ surface atomic composition, making it difficult to assign a specific chemistry for the investigated particles’ external layer. The influence of the surfactant makes the interpretation of high-resolution C 1s and O 1s core emission line spectra also quite problematic and not univocal. By extension, we expect the PVA surfactant to modify the unknown atomic concentration ratios, also in the case of 3P NP systems.

**Figure 4:**
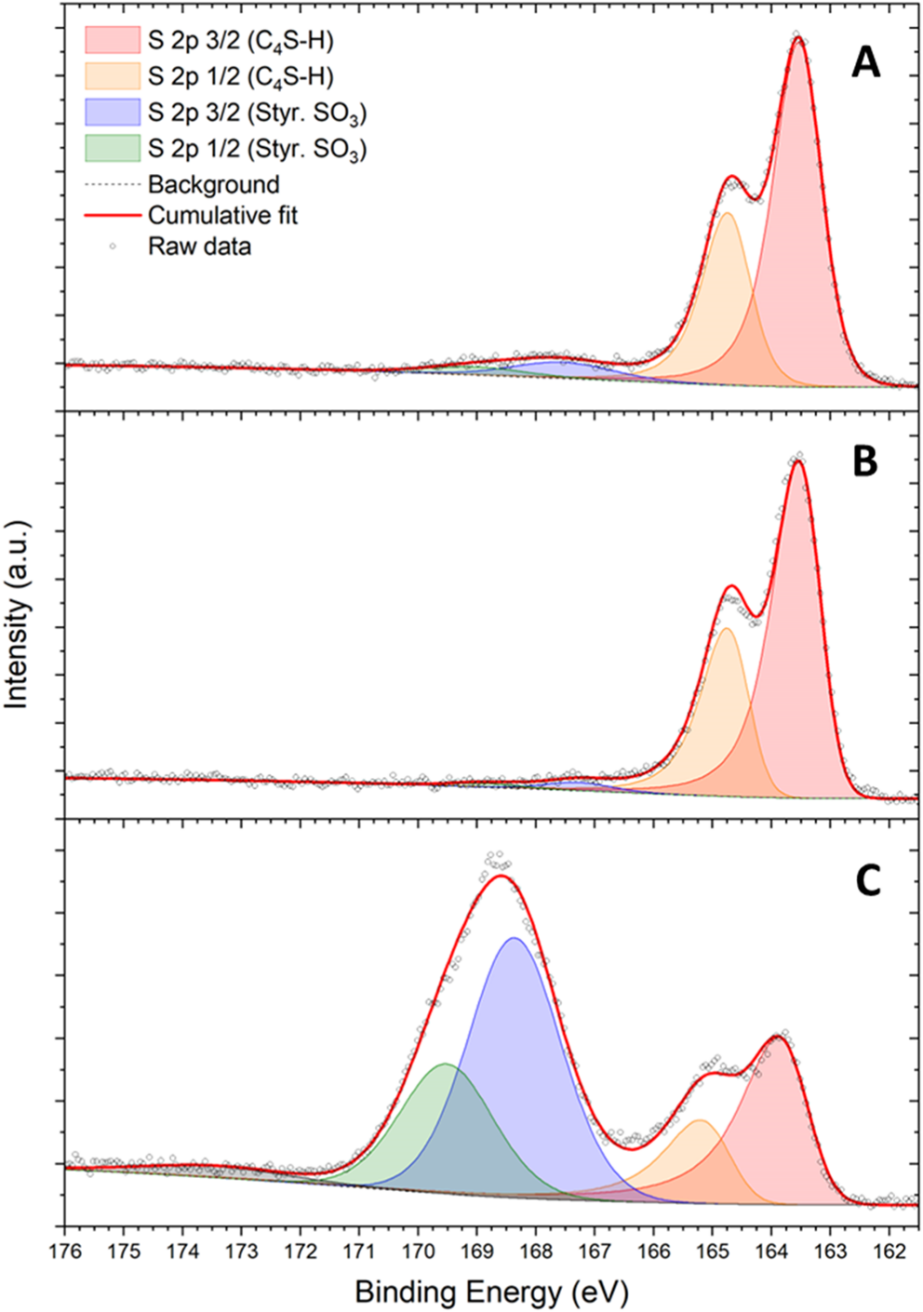
X-ray photoelectron spectroscopy signals of raw data (black circles) and fitted profiles (shaded peaks for individual components and red line for the cumulative fit) of the S 2p doublet structure of (a) 3P, (b) P3HT, and (c) PEDOT:PSS NPs.

More insights can be obtained by comparing the high-resolution spectral envelope of the S 2p photoelectron lines across different samples (Figure 4A–C), which are peculiar characteristics of P3HT and PEDOT:PSS but not of PVA. The S 2p core line shape for the 3P NP surface is shown in Figure 4A, while P3HT NPs and PEDOT:PSS controls are shown in Figure 4B,C, which clearly show differences in the surface composition. The assignments for thiophene sulfur (C_4_S–H) and styrenesulfonate (−SO_3_) doublets are already well described in other publications.^44,45^ Deconvolution of the complex spectral envelopes has been carried out on the basis of *a priori* knowledge of the chemical composition and the results obtained on reference surfaces. In all compounds, the observed S 2p 3/2 at 163.5 eV and S 2p 1/2 at 164.7 eV doublets are present and can be conveniently fitted (spin–orbit separation, 1.2 eV; intensity ratio, 1:2) with asymmetrical Lorentzian line shapes to reflect the effect of valence band electrons due to P3HT and/or PEDOT conductive characters.^46^ The thiophene sulfur (C_4_S–H) signal is present in all three samples. In the case of both 3P NPs and P3HT NPs, minor intensity contributions to the overall line shape are observed at a higher binding energy around 167.5 eV (less than 5% of total region intensity). Doublets in this region can be assigned to other sulfur atoms bound to more electronegative atoms; since the particles were manipulated in ambient air, it is possible that these are oxidized compounds (*e.g.*, sulfone group, −SO_2_) resulting from surface photo-oxidation.^47^ The PEDOT:PSS spectra in Figure 4C instead show two features with a comparable intensity. The low-binding-energy doublet can be fitted using the same asymmetrical doublet for thiophene sulfur (C_4_S–H), while the additional doublet at a higher energy shift, namely S 2p 3/2 at 168.4 eV and S 2p 1/2 at 169.6 eV, can be described using a pair of symmetrical functions. The observed energy shifts match well with reported values for sulfonate (−SO_3_), and the intensity ratio between the two doublets is ca. 2:1 in favor of the sulfonate group.

A comparison between 3P NPs and reference S 2p high-resolution spectra indicates that the surface of the NPs is mainly composed of P3HT molecules (based on the appearance of a thiophene sulfur doublet and the absence of a substantial sulfonate doublet). Intensity components in the higher binding energy region observed in 3P NP spectra are more intense with respect to the P3HT NP reference and are therefore attributable to oxidized sulfur functionalities arising from spurious surface oxidation effects, low concentration, and/or buried PEDOT:PSS.

Overall, optical (Figure 2), spectroscopic (Figure S1), and structural data (Figures 3 and 4) indicate the formation of a structured architecture within 3P NPs, with a preferential localization of PEDOT:PSS islets within the NP bulk and of P3HT at the NP surface exposed to the electrolyte environment. This architecture is expected to maximize the surface area available for charge dissociation and at the same time support the occurrence of photoactivated electron-transfer reactions, in particular of photoelectrochemical oxygen reduction processes.

### Electrochemical Characterization

**Figure 5:**
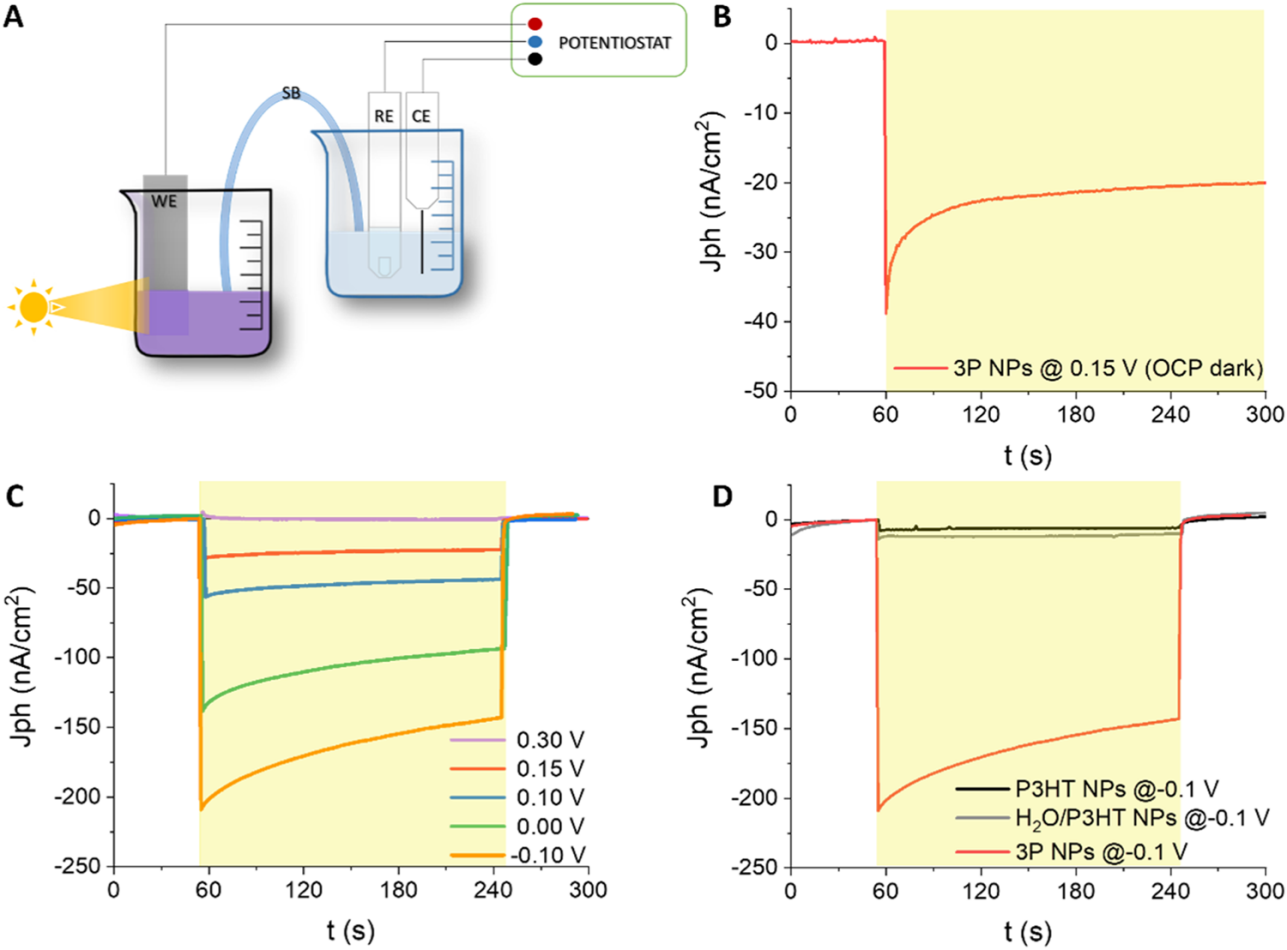
(A) 3-electrode photoelectrochemical setup for chronoamperometry (CA) recordings. (B) CA signal recorded with 3P NPs at the OCP in the dark (0.15 V *vs* RE); light is represented by the yellow shaded area. (C) CA signals recorded with 3P NPs under various electrical biases higher and lower than the OCP values (0.30, 0.15, 0.10, 0.00, and −0.10 V *vs* RE, respectively). (D) Comparison of CA signals recorded at −0.1 V in 3P NPs and relevant control systems, namely, H_2_O/P3HT NPs and P3HT NPs.

Optical, microscopic, and structural characterization presented above unequivocally demonstrate that 3P NPs are spatially self-organized in PEDOT:PSS multicores, surrounded by P3HT. The semiconductor is the predominant component at the outer interface with the electrolyte environment. To assess whether such architecture effectively turns into a more efficient charge separation and enhancement of photoelectrochemical reactions at the NP/water interface, we carried out photoelectrochemical (PEC) measurements in a 3-electrode configuration (Figure 5). NP dispersions in phosphate-buffered solution (PBS) were placed inside a PEC cell; an indium tin oxide (ITO) slab in direct contact with the NPs serves as the working electrode (WE), while the reference (RE) and the counter electrode (CE) are Ag/AgCl and platinum electrodes, respectively (Figure 5A).

The open-circuit potential (OCP) of the 3P NPs in the dark is around 0.15 V *vs* RE (OCP dark). Under continuous green light illumination (emission peak centered at 530 nm; power density, 1 mW/mm^2^) the OCP rises toward 0.35 V *vs* RE. Chronoamperometry (CA) experiments under potentiostatic control at the OCP dark potential show negligible dark current and buildup of a negative current signal at photoexcitation onset. After the initial transient, the photocurrent amplitude reaches a steady-state plateau value, amounting to about −20 nA/cm^2^, stable for 30 min upon continuous illumination (Figure 5B). CA shows a very stable photocurrent generation over time, which rules out any detrimental effect on the PEC performance of NPs at this time scale.

Figure 5C shows CA profiles recorded at several biases in the range +0.30 to −0.1 V *vs* RE, by employing 3P NPs. The increasing magnitude of the photocurrent (more negative) with increasing cathodic potentials *vs* OCP accounts for an enhancement of charge-carrier separation and a concomitant increase of electron transfer across the NP interface. A significant photocurrent of −150 nA/cm^2^ is obtained with the 3P NPs at the lowest potential imposed, *i.e.*, −0.1 V *vs* RE, which corresponds to 0.53 V *vs* RHE. This photocurrent value is about 1 order of magnitude higher than the one reported in the case of both P3HT (−6 nA/cm^2^) and H_2_O/P3HT NPs (−12 nA/cm^2^) control samples (Figure 5D).

CA data show that the 3P NP architecture, encompassing the presence of PEDOT:PSS multicores (Figure 3), the formation of an interphase between the P3HT component and the aqueous environment (Figure 4), and the improvement of P3HT crystallinity (as inferred from Figure 2), determines more efficient charge extraction, turning into sizably higher photocurrent generation. In line with previous works,^16,25,48^ the photocathodic current is due to the oxygen reduction reaction (ORR), whose final outcome is the formation of reactive oxygen species (ROS), such as superoxide ions and hydrogen peroxide. This process has been fully confirmed by photocurrent measurements within P3HT NP dispersion under deoxygenated conditions, as well as after reoxygenation.^16^ Precisely, the energy-band diagram for this interface supports the generation of superoxide ions having a standard redox potential reported as −0.33 V *vs* SHE,^49^ which lies close to the mid gap of P3HT frontier orbitals at the isolated materials.^25^ Moreover, such potential is thermodynamically accessible for a downhill electron transfer of photogenerated electrons populating the LUMO of the polymer when in contact with the electrolyte under illumination.

### 3P NPs/Endothelial Cell Biohybrid Interfaces

In brief, the 3P NP architecture enables localized ROS generation in a physiological-like environment upon visible-light excitation, which opens up the possibility to use them for redox medicine applications. Among other possibilities, we focus here on angiogenesis, as a valuable test bed for ROS physiological and therapeutic roles.^10^

**Figure 6:**
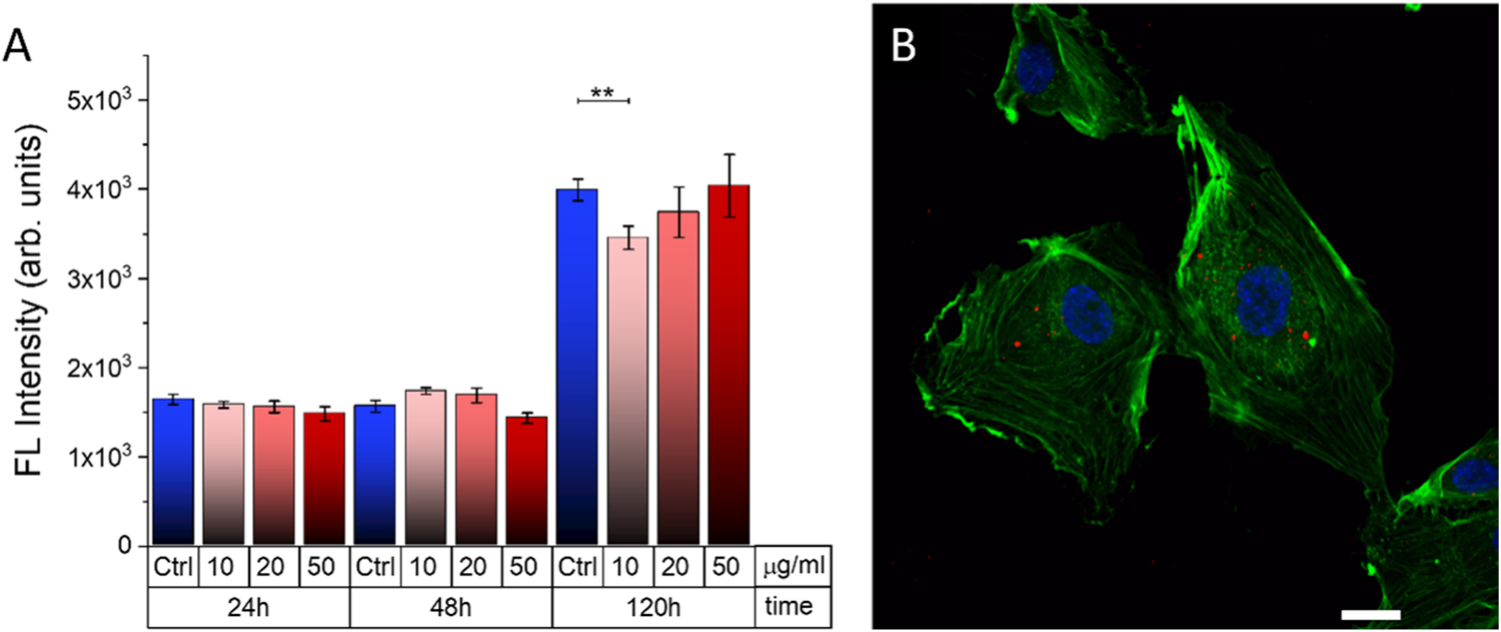
(A) Cell proliferation of HUVECs exposed to different concentrations of 3P NPs, at different time points after plating, evaluated as the fluorescence (FL) of the reduced form of the alamarBlue cell-viability reagent. Error bars represent the standard error of the mean (SE). Statistical significance was evaluated by Student’s *t* test (**p < 0.01). Not statistically significant differences among the different groups are not reported in the graphs. (B) Representative confocal image depicting HUVECs treated with 3P NPs. Cells were stained with Hoechst 33342 (nuclei, blue) and CellMask Green (membrane, green); NP fluorescence emission is in red. Scale bar, 20 μm.

In order to investigate the impact of different P3HT-based NPs on angiogenesis, we employed human umbilical vein endothelial cells (HUVECs), a well-established model for the study of the endothelium function.^50^ The excellent biocompatibility of bare P3HT NPs has been widely reported for several cell types and animal models.^3,9,13,16,51^Figure 6A shows the proliferation of HUVECs treated with 3P NPs. The cell cultures were incubated with the NP dispersions at different concentrations (10, 20, and 50 μg/mL) for 24 h. Cell proliferation was then studied at three different time points (24, 48, and 120 h), until a cell confluence of about 80% is reached, by using the consolidated alamarBlue assay. The latter provides a reliable indication of the metabolic activity of cultured cells and is a direct proof of cell proliferation capability. Cell cultures treated with 3P NPs at the highest concentration (50 μg/mL) do not show statistically significant differences in the proliferation rate with respect to control, untreated samples, and up to 5 days after plating. Confocal microscopy allows identification of the presence of NPs, internalized within the HUVEC cytosol (Figure 6B). Optical sections, with an incremental step of 150 nm, were measured to determine whether the NPs are localized within the cytosol or they are anchored to the external side of the cellular membrane (Figure S3). Figure 6B depicts a representative confocal image of HUVECs, 24 h after incubation with NPs, acquired at a z plane corresponding to the inner part of the cells. The appearance of red emission spots in the same plane of the actin filaments stained by phalloidin (green emission) suggests that the NPs have been efficiently internalized. Importantly, they are distributed around the perinuclear region and do not cross the nuclear membrane.

### Intracellular ROS and Ca^2+^ Measurements

The effective internalization of 3P NPs within HUVECs, while maintaining unaltered cell proliferation, opens up the opportunity to exploit their optical properties for local modulation of cell functions. In particular, the engineered architecture of 3P NPs, showing intermixed phases between the electron donor (P3HT) and electron acceptor (PEDOT:PSS) materials, is promising for controlling photocatalytic interfacial processes and subsequent effective modulation of intracellular ROS production. The ROS concentration, spanning over several orders of magnitude, plays a key role in many physiological processes,^19^ including metabolism regulation,^52^ immune system control,^53^ blood pressure modulation,^54^ neurotransmission,^55^ and angiogenesis.^10^ In addition, several pathological conditions implicate alterations in ROS physiology, orienting research efforts toward innovative therapeutic methods for the *in situ* modulation of ROS.^56^ On the other hand, excess ROS (approximate extracellular concentration of above 10 uM, depending on the specific cell type) can lead to cell apoptosis and cell death. Therefore, it is of critical importance to develop new tools for precise ROS control, depending on the targeted applications, either in the eustress or distress regime.^19,55,57^

Our previous reports demonstrated the possibility of employing the P3HT polymer, either in the form of thin films or as NPs, to optically modulate the intracellular ROS concentration and the intracellular redox balance in human embryonic kidney cells (HEK-293),^16^ HUVECs,^13,15^ endothelial colony-forming cells (ECFCs),^11,57^ and in a cardiac cell model (HL1).^14^

**Figure 7:**
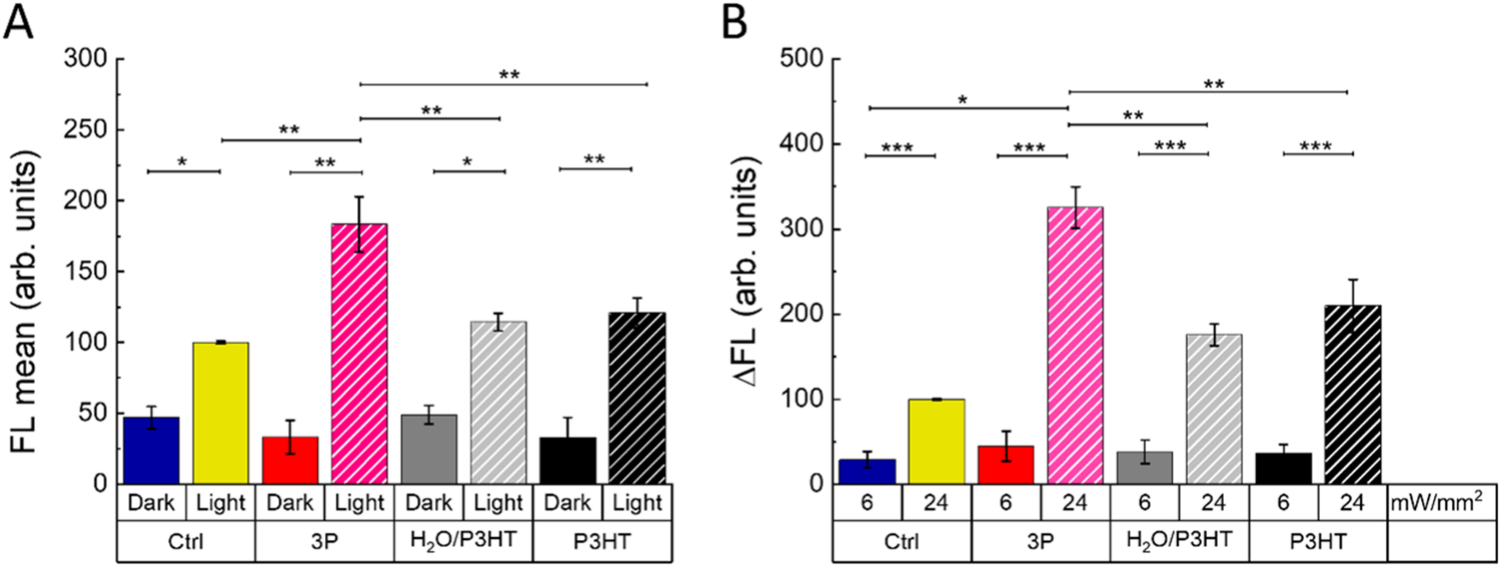
(A) Intracellular ROS production, evaluated by the measurement of the H_2_DCF-DA probe fluorescence intensity (FL), in NPs-untreated and -treated HUVECs, both in the dark and exposed to the photoexcitation protocol. Data are represented as mean ± SE values over statistical samples of *n* = 88 (control), *n* = 96 (3P NPs), *n* = 79 (H_2_O/P3HT NPs), and *n* = 81 (P3HT NPs), where *n* represents the number of cells over three different experimental replicas for each condition. (B) Relative fluorescence increase (ΔFL) of the Fluo-4 calcium indicator. Data are shown as mean ± SE over statistical sample sets of *n* = 78 cells (control), 76 (3P NPs), 69 (H_2_O/P3HT NPs), and 73 (P3HT NPs). Data have been averaged over three biological replicas for each condition. In both panels, statistical significance has been evaluated by one-way ANOVA analysis followed by a post hoc Tukey test. *P*-values of the test are assigned as follows: *** for *p* < 0.001, ** for *p* < 0.02 and * for *p* < 0.05. Not statistically significant differences among the different groups are not reported in the graphs.

Herein, we critically evaluated the impact of the composite structure on intracellular ROS production by employing two ROS fluorescent probes, namely, 2,7-dichlorodihydrofluorescein diacetate (H_2_DCF-DA, Figure 7A) and aminophenyl fluorescein (APF, Figure S4). H_2_DCF-DA is sensitive to a large variety of ROS, including H_2_O_2_, HO^•^, and ROO^•^.^16^ Bare P3HT and H_2_O/P3HT NPs were used as controls to evaluate the contribution of PEDOT:PSS in ROS generation. Figure 7 shows that cells treated with both NPs and light-excitation protocol (excitation wavelength peak, 470 mm; illumination duration, 3 min CW light; photoexcitation density, and 85 mW/mm^2^) present a statistically significant increase in ROS production, as compared to control, untreated cells, and NP-treated cells not exposed to photoexcitation. Importantly, 3P NPs show ∼+40% relative increase as compared to NPs without the PEDOT:PSS component.

The experiment was repeated with an APF intracellular ROS probe, which provides complementary information to H_2_DCF-DA, given its enhanced sensitivity to HO^•^, ONOO^–^, and HOCl.^58^ The results obtained (Figure S4) are in line with the ones obtained with H_2_DCF-DA results, showing a statistically significant increase of intracellular ROS, with respect to the untreated sample, of about +35%.

Fine-tuning of cell signaling processes was regulated by a tight interplay between the ROS concentration and Ca^2+^ ion signaling, as widely reported in the literature.^57,59,60^ In particular, it has been reported that in endothelial cells, functional crosstalk between Ca^2+^ and ROS is in place, wherein intracellular ROS elicit endothelial Ca^2+^ signals by regulating inositol-1,4,5-trisphosphate receptors, sarco-endoplasmic reticulum, two-pore channels, store-operated Ca^2+^ entry, and several isoforms of transient receptor potential channels. In parallel, multiple vascular functions are regulated by ROS-induced endothelial Ca^2+^ signals.^61^

This evidence prompted us to investigate the effect of 3P NPs on HUVECs’ intracellular Ca^2+^ dynamics, studied using fluorescence microscopy experiments and the Fluo-4 AM calcium-sensitive probe. Since the Fluo-4 excitation spectrum partially overlaps with the P3HT optical absorption, the contribution of the polymer photostimulation cannot be completely disentangled. Therefore, we tuned the light-excitation density to identify a threshold at which the optical modulation of Ca^2+^-transient dynamics is minimized (namely, 530 nm, 3 min, 6 mW/mm^2^). In fact, the NP-treated cells stimulated with this protocol showed intracellular calcium signals that are fully comparable to those registered in NP-untreated controls (Figure 7B). Under more intense photoexcitation conditions (530 nm, 3 min, 24 mW/mm^2^), a significant increase in Ca^2+^ transients is obtained instead, showing the highest relative percentage increase in the case of 3P NPs (about +225, +85, and +55% compared to untreated control, for 3P, H_2_O/P3HT, and P3HT NPs, respectively).

### Tubulogenesis Assay

The results presented above unequivocally demonstrate that PEDOT:PSS enhances the photoelectrochemical efficiency of the NPs toward intracellular ROS production and their efficacy in optical modulation of Ca^2+^ dynamics in HUVECs. A highly interesting, potential application is optical modulation of angiogenesis. This is in fact crucial for organ growth and repair, as well as in many pathological conditions, including ischemic heart disease, cancer, and ocular and skin disorders;^62^ however, its effective modulation in a drug-free, temporally controlled, and noninvasive manner represents a currently unmet therapeutic target. Significantly to the present work, several recent literature reports demonstrate the key role played by ROS and Ca^2+^, in synergy, in this process.^10,57,60,62,63^

**Figure 8:**
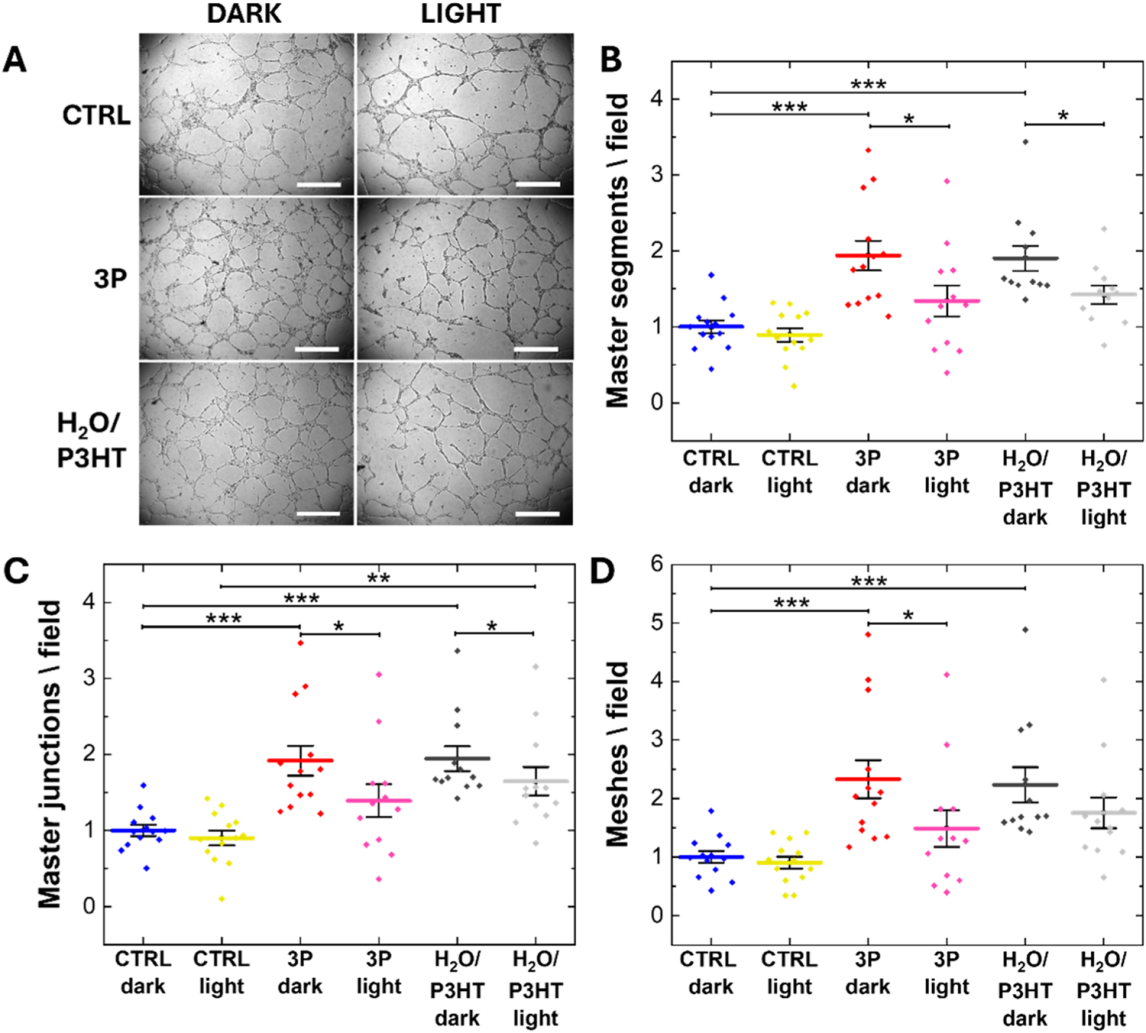
(A) Representative bright-field images depicting the capillary-like network formed by HUVECs during the *in vitro* angiogenesis assay in all of the considered conditions. Scale bars, 500 μm. Average number of master segments (B), master junctions (C), and meshes (D) obtained from the quantitative analysis of the bright-field images acquired 6 h after plating on Geltrex. The results are represented as the mean ± SE of three different experiments. Data were compared with the Mann–Whitney nonparametric test. *p < 0.05, ***p < 0.001.

We carried out a tube formation assay that recapitulates many steps of the angiogenic process, including adhesion, migration, and tubule formation,^11^ by plating HUVECs, loaded with NPs, on a reconstituted basement membrane extracellular matrix (Geltrex). The latter mimics the membrane that surrounds the blood vessels *in vivo*, and it is necessary for cellular assembly into bidimensional capillary-like networks *in vitro*. We studied the morphology of the network through optical microscopy (Figure 8A) after exposing the cells to a long-term photoexcitation protocol (central excitation wavelength, 530 nm; photoexcitation density, 6 mW/cm^2^; light stimuli duration, 100 ms; repetition rate, 1 Hz; overall protocol duration, 6 h) or leaving them in the dark. We provide a quantitative analysis of the cell network development by considering three main morphological features, namely: (1) number of master segments, *i.e.*, tubes that link different regions of the network from both sides (Figure S5, yellow segments), (2) number of master junctions, *i.e.*, junctions that connect at least three master segments (Figure S5, pink circles), and (3) number of meshes, *i.e.*, closed regions delimited by master junctions and master segments (Figure S5, cyan loops).^11,64^ HUVECs incubated with both 3P NPs and H_2_O/P3HT NPs, in the dark, give rise to extended capillary-like networks, characterized by a higher number of master junctions, master segments, and meshes with respect to cells not incubated with NPs (CTRL dark, Figure 8B–D). The cells subjected only to light stimulation (CTRL light) do not show significant differences from the CTRL dark case (Figure 8B–D). Instead, the photoexcitation coupled with both NP types leads to a statistically relevant decrease of the pro-angiogenic effect, as compared to the corresponding NP-treated cases in the dark (Figure 8B–D). However, this light-induced reduction of the network features is significantly higher in the presence of PEDOT:PSS as compared to the H_2_O/P3HT case (decrease percentage light *vs* dark: master segments, 31 and 18%; master junctions, 28 and 15%; meshes, 36 and 22% for 3P and H_2_O/P3HT NPs, respectively). The results highlight that the coupling of the P3HT active layer with PEDOT:PSS multicores enhances the physiological outcome triggered by light and NPs.

The use of exogenous nanosized materials has been recently proposed for the modulation of the angiogenic process. Organic and inorganic NPs displayed both antiangiogenic and pro-angiogenic properties, through the targeting of different angiogenic pathways.^10,63^ Red-light excitation of a low band-gap conjugated polymer (PTB7) triggers an angiogenic effect in a minimally invasive manner and with high spatial resolution.^12^ Interestingly, with P3HT-based NPs, we reported a bimodal pro-/antiangiogenic behavior, similar to the one observed with PTB7 NPs. In most cases, the NP-triggered angiogenesis modulation was related to intracellular ROS generation, and the observed effects were dose-dependent. In particular, the low level of ROS acts as a pro-angiogenic signaling molecule, whereas the high level of ROS acts as an inhibitor for angiogenesis.^10^ This is in line with our results, showing the highest light-induced antiangiogenic effect with the 3P NP system (Figure 8), deterministically related to the highest intracellular ROS variation and increased intracellular Ca^2+^ concentration (Figure 7).

## Conclusions

Biocompatible, composite 3P NPs based on PEDOT:PSS conducting multicores dispersed within a green light-absorbing semiconducting P3HT polymer have been realized. The introduction of a diffused interface between the two components leads to sizable improvement of the charge dissociation, thus considerably enhancing, by about +40%, the efficiency toward photoelectrochemical oxygen reduction and production of ROS within the cell cytosol, as compared to bare semiconducting NPs without PEDOT:PSS. Subsequently, the presence of conducting islets has a major physiological outcome, with a relative increase of about 50% in Ca^2+^-signaling activity as compared to bare P3HT NPs. Importantly, this allows us to significantly broaden the dynamic range of photoexcitation densities necessary for effective NP activation, *i.e.*, to substantially lower the light intensity while maintaining the NP concentration unaltered, or, vice versa, to reduce the NP doses, while maintaining the light intensity at a fixed value.

Among many other possibilities, 3P NPs find an interesting application in therapeutic/tumoral angiogenesis treatment, where a tiny change in the delicate intracellular redox balance, closely interconnected with ROS production and Ca^2+^ signaling, can easily determine the shift from a eustress condition toward distress. In fact, we demonstrate that 3P NPs enable a significant modulation of several quantitative parameters related to the pro- *vs* antiangiogenic condition, by a factor of about 2, as compared to NPs without PEDOT:PSS.

In brief, enhancement of photoelectrochemical efficiency of light-sensitive polymer NPs allows for a more precise tuning of the light dose–response effect, thus opening interesting perspectives for all those *in vivo* applications of NP optical transducers, in which the NP concentration and light power density must be carefully balanced. Toward this goal, novel chemically modified conductive polymers, characterized by superior stability, good water dispersibility, and mixed ionic–electronic conductivity, open interesting perspectives for further implementation of the approach.^70,71^ Besides angiogenesis, 3P NPs may be usefully employed as injectable artificial photoreceptors, as intracellular phototransducers in regenerative medicine, as multifunctional tools for photoactuation, drug delivery, and bioimaging, as well as in tissue engineering for modulation of the intracellular redox balance and for optical control of the cell fate, comprising key processes like proliferation, migration, differentiation *vs* inflammation, growth arrest, and cell death.

## Materials and Methods

### Synthesis of Poly(3-hexylthiophene-2,5-diyl)-Based NPs

Poly(3,4-ethylenedioxythiophene):poly(4-styrenesulfonate) (PEDOT:PSS)/P3HT NPs (3P NPs) were fabricated by a modified double emulsion method. P3HT (regioregular, average *M*_w_: 20,000–45,000, Sigma-Aldrich) was dissolved in chloroform (CHCl_3_, Sigma-Aldrich) at a concentration of 10 mg/mL. The solution was sonicated for 15 min and stirred at 60 °C overnight. The resulting solution was filtered using a 0.2 μm PVPDF syringe filter to eliminate aggregates. PEDOT:PSS 3.0–4.0% in H_2_O (Sigma-Aldrich) was first diluted with deionized water in a 1:2 ratio, then 200 μL of this dispersion was added to 5 mL of P3HT/CHCl_3_ solution. The resulting mixture was ultrasonicated with a tip sonifier (Branson Digital Sonifier 450 (400 W), equipped with a 13 mm step horn and a flat tip) for 3 min at 30% amplitude under a pulsed protocol (20 s ON/10 s OFF) to obtain the first emulsion. The second and final emulsion was obtained by adding the first emulsion to 25 mL of 0.2% PVA in H_2_O solution and ultrasonicating with the tip sonifier for 4 min at 40% amplitude (pulsed protocol, 20 s ON/10 s OFF). The ultrasonication processes were performed by cooling the mixture in an ice bath to avoid excessive heating. The double emulsion was placed in a rotavapor at 40 °C for 20 min to evaporate the organic solvent. Finally, the double emulsion was stirred at 40 °C for 3 h to ensure the complete evaporation of CHCl_3_. Upon evaporation of the solvent, the 3P composite particles were formed. H_2_O/P3HT NPs were synthesized by following the same procedure adopted for 3P NPs by substituting the PEDOT:PSS solution with ultrapure H_2_O. P3HT NPs were obtained by a single emulsion method, by mixing the P3HT/CHCl_3_ solution with 0.2% PVA in H_2_O (1:5 volume ratio) and following the same processes of ultrasonication and CHCl_3_ evaporation adopted in the case of 3P NPs.

### Morphological and Optical Characterization

Prior to SEM experiments, NP suspensions (100 μL) were deposited by drop-casting on oxygen plasma-treated Si substrates, allowing solvent evaporation in air at room temperature. All SEM micrographs were acquired by using a ZEISS GeminiSEM 560 scanning electron microscope (operating voltage, 5 kV; working distance, 4 mm) equipped with a secondary electron detector.

The average particle size was evaluated by the dynamic light scattering (DLS) technique using a Zetasizer Nano ZS (Malvern Instruments, U.K.). The particle sizes were obtained by averaging 16 measurements (3 cycles for each run at an angle of 173° at 25 °C). The laser wavelength was 633 nm, and measurements were performed in disposable plastic cells.

UV/vis spectra were acquired with a PerkinElmer Lambda 1050 spectrophotometer.

### Raman Measurements

Raman spectroscopic analysis was conducted using a MonoVista CRS S&I spectrometer, which was equipped with a red laser source emitting light at a wavelength of 785 nm. To safeguard the samples from photodamage, the laser power was attenuated to approximately 2.7 mW. The acquisition of Raman spectra was accomplished using a 10× objective with a numerical aperture (NA) of 0.30 and a spectral resolution of 3 cm^–1^. The collected measurements were baseline-corrected and normalized to the intensity of the band at 1385 cm^–1^ to derive the difference profile between 3P NPs and H_2_O/P3HT NP Raman spectra.

### Small-Angle X-ray Scattering Measurements

SAXS data were acquired on the Austrian SAXS beamline^65^ at ELETTRA Synchrotron (Trieste, Italy) at a photon energy of 8 keV, corresponding to a wavelength of 0.154 nm. The sample to detector distance was set to 1499.018 mm, covering a *q* range between 0.095 and 4.86 nm^–1^, with *q* = 4*p* sin θ/λ, where λ is the wavelength and 2θ is the scattering angle. The angular scale of the measured intensity was calibrated using silver behenate (CH_3_–(CH_2_)_20_–COOAg, characterized by a *d*-spacing value of 58.38 Å). The two-dimensional (2D) SAXS patterns were acquired using a PILATUS3 X 1M detector (Dectris Ltd., Baden, Switzerland). The 2D images were integrated to one-dimensional (1D) images using SAXSDOG,^66^ the data reduction pipeline available at the Austrian SAXS beamline, and analyzed using IGOR Pro (Wavemetrics, Inc., Lake Oswego, OR). SAXS acquisitions were performed on the 3P NPs synthesis solution at two different time points: (i) just after the formation of the second emulsion and (ii) after CHCl_3_ complete evaporation. Scattering patterns were fitted by using the same approach proposed by Pedersen and co-workers,^33^ where the forwarded scattering probability can be written as the sum of three components: *I*(*q*) = *I*(*q*)*D* + *I*(*q*)_TS_ + *I*(*q*)_P_. Here, *I*(*q*)_D_ represents the diffraction peak following the Gaussian distribution. *I*(*q*)_TS_ = *C*/(*a*_2_ + *c*_1_ + *c*_2_) is the model proposed by the Teubner–Strey model,^43^ where *a*_2_ (>0), *c*_1_ (<0), and *c*_2_ are the coefficients from which domain correlation length (ξ) and interdomain distance (*D*) have been, respectively, calculated as 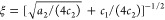
and 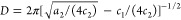 Finally, *I*(*q*)_P_ represents a core–shell form factor, where the outer radius of the particle follows the Schultz distribution;^67^ as input parameters from the particle size (as the average particle size exceeds the experimental resolution), results from DLS were used during data fitting.

### X-ray Photoelectron Spectroscopy

XPS measurements were performed with an Axis Ultra spectrometer (Kratos Analytical, Manchester, U.K.), using a K_α_ Al monochromatic source (*h*ν = 1486.6 eV) operating at 150 W (15 kV, 10 mA) and an X-ray spot size of 100 × 100 μm^2^ in hybrid electromagnetic lens configuration mode. The residual pressure of the analysis chamber during the analysis was less than 8 × 10^–9^ Torr. For each sample, both survey spectra (0–1200 eV; pass energy: 80 eV) and high-resolution spectra (pass energy: 40 eV) were recorded. The surface charge was compensated by an electron flood gun system, and the energy scale was calibrated by setting the C 1s hydrocarbon peak to 285.00 eV in binding energy. The data were acquired using Vision2 software (Kratos Analytical, Stretford, U.K.), and the analysis of the XPS peaks was carried out using a commercial software package (CasaXPS version 2.3.18PR1, Casa Software, Ltd., Teignmouth, U.K.). Peak fitting was performed with no preliminary smoothing. Asymmetric and symmetric Voigt (70% Gaussian and 30% Lorentzian) functions were used to approximate the experimental line shapes of the fitting components after a three-parameter Tougaard-type background subtraction. NP samples dispersed in Milli-Q water were drop-cast on both clean Teflon substrates. The use of Teflon substrate allows minimizing the uncertainties due to adventitious hydrocarbon contamination, as already described in previous work.^68,69^

### Chronoamperometry Measurements

Photocurrent measurements were carried out by using a potentiostat/galvanostat (Autolab, PGSTAT 302N). The electrochemical cell was in a three-electrode configuration and was divided into two compartments, connected by a saline bridge. One compartment contained the reference and the counter electrodes of a KCl-saturated Ag/AgCl and a Pt wire, respectively, immersed in the pure electrolyte (10 mM phosphate buffer, pH 7). The second compartment contained the indium tin oxide working electrode (Xinyan Technology, 15 nm thickness, 15 ohm/sq sheet resistance) and the P3HT-based NPs dissolved in the same electrolyte solution. Chronoamperometry measurements were performed at electrochemical equilibrium, by applying a potential equal to the open-circuit potential (OCP), and by changing the applied voltage in the range (OCP: 250 mV) ≤ V ≤ OCP + 150 mV *vs* Ag/AgCl. A continuous light source (Thorlabs LED M470L3-C5, 470 nm central emission wavelength) was used for the photoexcitation, with a power density of 2.7 mW/mm^2^.

### Cell Culture

Human umbilical vein endothelial cells (HUVECs), purchased from Promocell, were grown in endothelial cell growth medium 2 (Promocell) supplemented with fetal calf serum (0.02 mL/mL), recombinant human epidermal growth factor (5 ng/mL), recombinant human basic fibroblast growth factor (10 ng/mL), recombinant human insulin-like growth factor (LONG R3 IGF, 20 ng/mL), recombinant human vascular endothelial growth factor 165 (0.5 ng/mL), ascorbic acid (1 μg/mL), heparin (22.5 μg/mL), hydrocortisone (0.2 μg/mL), streptomycin (100 U/mL), and penicillin (100 U/mL). The cells were maintained in T-75 culture flasks, pretreated with a gelatin solution, and kept at 37 °C in 5% CO_2_. After reaching 80–90% confluence, cells were detached by incubation with 0.5% trypsin–0.2% EDTA (Sigma-Aldrich) for 5 min and plated on glass substrates for experiments. The glass coverslips were pretreated with fibronectin (from bovine plasma, 2 mg/mL in PBS, Sigma-Aldrich) for 30 min to promote cell adhesion.

### Proliferation Assay

HUVECs were plated in 12-well plates by employing cell growth medium without phenol red. Cell proliferation was evaluated after 24, 48, and 120 h of incubation with 3P NPs. Prior to measurements at each time point, the growth medium was replaced with fresh medium containing 100 mg/mL alamarBlue (Thermo Fisher). The alamarBlue reagent is based on resazurin, a cell-permeable nonfluorescent compound that upon entering living cells is reduced to the highly fluorescent resorufin. The fluorescence of the latter is thus an indicator of the viability and proliferation of the cells. The samples were incubated for 3 h at 37 °C under 5% CO_2_ in the dark. Then, three aliquots of culture media (100 μL) were placed in black 96-well microplates, and their fluorescence was acquired using a TECAN Spark microplate reader (excitation wavelength: 530 nm; emission wavelength: 590 nm).

### Immunofluorescence Analysis

The cells grown on fibronectin-coated glass coverslips were washed twice with PBS and fixed for 20 min at RT in 4% paraformaldehyde and 4% sucrose in 0.12 M sodium phosphate buffer, pH 7.4. The fixed cells were preincubated for 20 min in gelatin dilution buffer (GDB: 0.02 M sodium phosphate buffer, pH 7.4, 0.45 M NaCl, 0.2% (w/v) gelatin) containing 0.3% (v/v) Triton X-100 and subsequently incubated with Alexa Fluor 488 Phalloidin conjugated in GDB for 1 h at RT and finally washed with PBS and incubated for 5 min with 1 μM DAPI in PBS. The confocal images were acquired using a Nikon Eclipse Ti2 with a 60× objective.

### Intracellular Reactive Oxygen Species and Ca^2+^ Detection

Dichlorofluorescein diacetate (H_2_DCF-DA, Sigma-Aldrich) and 2-[6-(4′-amino)-phenoxy-3*H*-xanthen-3-on-9-yl]benzoic acid (APF, Sigma-Aldrich) were employed for the intracellular detection of reactive oxygen species (ROS). 3P, H_2_O/P3HT, and pristine P3HT NPs were administered to the cells 3 h after the cell plating in Krebs-Ringer HEPES-buffered (KRH) extracellular medium (5 mM HEPES, 135 mM NaCl, 5,4 mM KCl, 1 mM MgCl_2_, 1.8 mM CaCl_2_, and 10 mM glucose, all purchased from Sigma-Aldrich; pH 7.4) at 20 μg/mL concentration. After 24 h of NP addition, HUVECs were rinsed with KRH to remove not internalized NPs. Under light conditions, samples were subsequently photoexcited for 3 min with a LED system (Lumencor Spectra X light engine; λ = 470 nm, 85 mW/mm^2^) fiber-coupled to an inverted microscope (Nikon Eclipse Ti) equipped with a 20× objective. Subsequently, the cells were incubated with H_2_DCF-DA and APF for 30 and 40 min, respectively. After carefully washing out the excess of the probe from the extracellular medium, DCF and APF fluorescence were recorded (excitation/emission wavelengths, 490/520 nm) with an inverted microscope (Nikon Eclipse Ti), equipped with a 20× objective and an sCMOS camera (Prime BSI, Teledyne Photometrics; Tucson, Arizona).

Prior to Ca^2+^ imaging recordings, HUVECs were loaded for 30 min at 37 ^◦^C with a 1 μM Fluo-4 calcium-sensitive probe (Life Technologies) in KRH solution. Then, the cells were washed for 10 min with prewarmed KRH solution before recordings. Photoexcitation and Fluo-4 fluorescence acquisition were carried out with the same light source and microscope employed for ROS recordings (photoexcitation conditions: illumination duration: 3 min; emission peak wavelength: 485 nm; photoexcitation density, 6 and 24 mW/mm^2^). In the case of both ROS and Ca^2+^ measurements, the variation of fluorescence intensity was evaluated over regions of interest (ROIs) covering single-cell areas, and reported values represent the average over multiple cells. See the caption of Figure 7 and Supporting Information for additional details about statistical analysis. Image processing and data analysis were carried out with ImageJ and Origin 2018 software, respectively.

### Tubulogenesis Assay

HUVECs were cultured as described in the “Cell Culture section” and plated on top of a bare glass. After 3 h of plating, the cells were treated with different types of NPs at 20 μg/mL concentration in culture medium. After 20 h of incubation with NPs, the cells were detached, resuspended, and plated on 96-well plates pretreated with 90 μL of Geltrex LDEV-Free Reduced Growth Factor Basement Membrane Matrix (Thermo Fisher) at 3 × 10^4^ cells/cm^2^. The plates were maintained at 37 °C and 5% CO_2_ and optically excited with LED light sources (central excitation wavelength: 530 nm, 6 mW/cm^2^) or left in the dark. The capillary network formation was assessed 6 h later by acquiring bright-field images with an inverted microscope (NIKON Eclipse Ti) equipped with a 4× objective. The quantification of the main features of the capillary-like network was performed by employing the Angiogenesis Analyzer plug-in of ImageJ. Mean values were averaged over 12 fields of view belonging to 3 independent experimental sessions.

## Supporting Information Available

Raman characterization; XPS survey spectra; confocal imaging Z-stacks; intracellular ROS imaging using an APF probe; and representative graphical representation of the detected and vectorized components of the HUVEC capillary-like network (PDF)
