# Supplementary material for: Composite Thiophene-Based Nanoparticles: Revisiting the PEDOT:PSS/P3HT Interface for Living-Cell Optical Modulation
Source: ACS Appl Mater Interfaces. 2025 Apr 4;17(15):22434–47. doi: 10.1021/acsami.5c02115 (PMC12012720; doi:10.1021/acsami.5c02115)
Supplement: Supplementary file 1 — am5c02115_si_001.pdf [file am5c02115_si_001.pdf]

Supporting Information

Composite thiophene-based nanoparticles: revisiting the PEDOT:PSS/P3HT interface for living cell optical modulation

Gabriele Tullii[1,§,*], Christian Bellacanzone[1,§], Hansel Comas Rojas[1], Francesco Fumagalli[2], Carlotta Ronchi[1], Anthea Villano[1,3], Federico Gobbo[1], Marco Bogar[4], Barbara Sartori[5], Paola Sassi,[6] Giulia Zampini[6], Giulia Quaglia[6], Loredana Latterini[6], Heinz Amenitsch[5], Maria Rosa Antognazza[1,*]

[1]*Center for Nano Science and Technology, Istituto Italiano di Tecnologia, Via Rubattino 81, 20134 Milano, Italy*

[2] *European Commission, Joint Research Centre (JRC), 21027 Ispra, Italy*

[3]*Politecnico di Milano, Physics Dept., P.zza L. da Vinci 32, 20133 Milano, Italy*

[4]*Department of Engineering and Architecture, University of Trieste, Via Alfonso Valerio 6/1, 34127 Trieste, Italy*

[5]*Graz University of Technology, Institute of Inorganic Chemistry, Stremayrgasse 9/4, A-8010 Graz, Austria*

[6] *Dipartimento di Chimica, Biologia e Biotecnologie, Università di Perugia, Via Elce di Sotto, 8, 06123 Perugia, Italy*

[§]*Equally contributing authors*

[*]*Corresponding authors, Gabriele.tullii@iit.it; mariarosa.antognazza@iit.it*

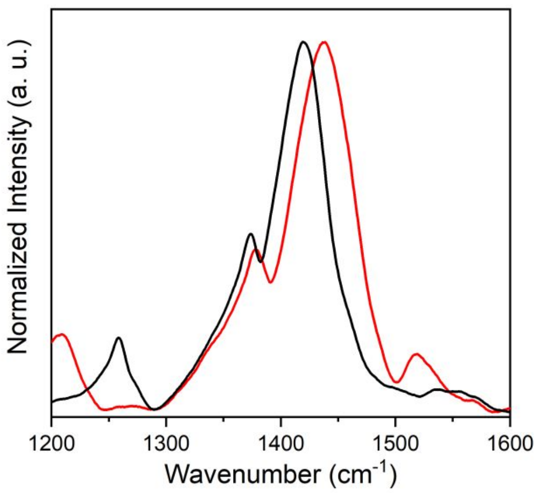

Figure S1. Raman spectra of the difference between the 3P NPs and control H_2O/P3HT NPs (red line), and bare PEDOT:PSS NPs (black line).

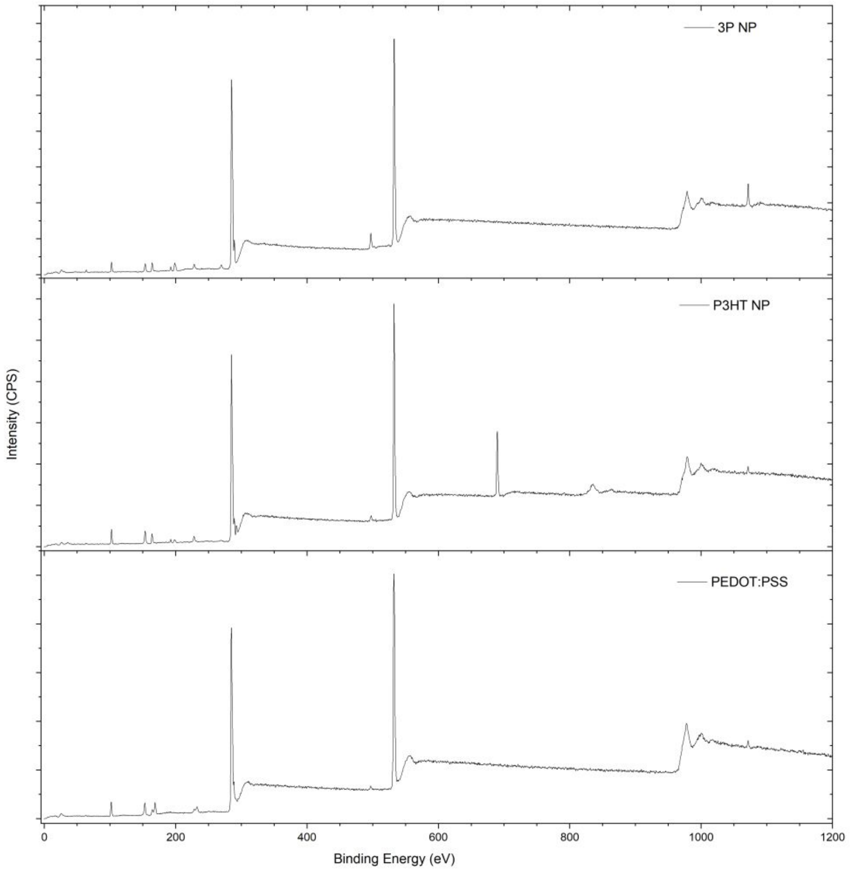

Figure S2. XPS survey spectra showing both photoelectron and auger emission lines from 3P NP/PVA, P3HT NP/PVA and PEDOT:PSS samples deposited on a Teflon C_2F_4 substrate. Photoelectron lines C 1s, O 1s, S 2p, Si 2p, Na 1s, Cl 2p and F 1s were used for semiquantitative elemental analysis.

	O/C meas.	O/C stoich.	S/C meas.	S/C stoich.
3P NP/PVA	0.31 [12%]	n.a.	0.02 [30%]	n.a.
P3HT NP/PVA	0.43 [32%]	0.00	0.03 [23%]	0.10
PEDOT:PSS [1:1]	0.35 [3%]	0.36	0.04 [3%]	0.14
PVA	0.52 (*)	0.50	0.00 (*)	0.00

n.a.: not applicable, surface composition unknown.

Table S1. Atomic concentration ratios of oxygen and sulphur over carbon for the 3P NP/PVA, P3HT NP/PVA and PEDOT:PSS samples. Both experimentally measured and stoichiometric values are indicated in separate columns. Relative standard deviation is indicated in square brackets where applicable. (*) Values taken from: Pierre Louette, Frederic Bodino, and Jean-Jacques Pireaux, "Poly(vinyl alcohol) (PVA) XPS Reference Core Level and Energy Loss Spectra" Surface Science Spectra 12, 106 (2005); doi: 10.1116/11.20050922

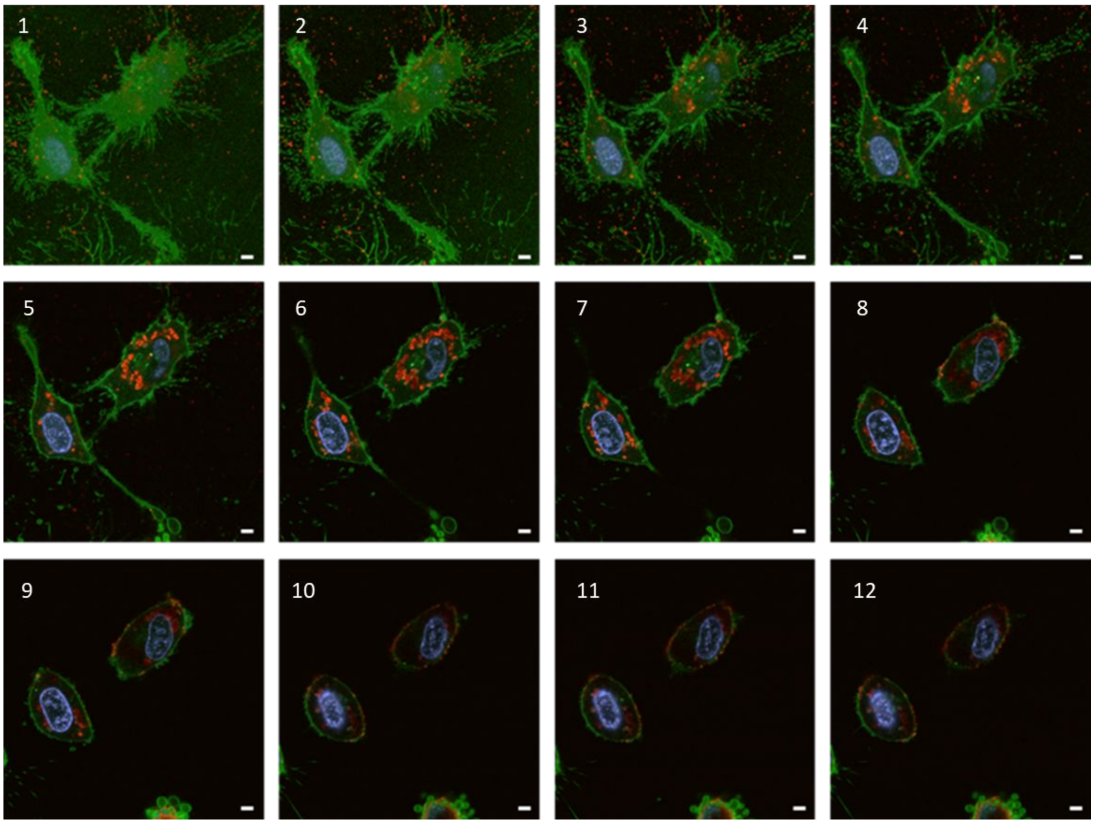

Figure S3. Confocal optical sections showing HUVECs treated with 3P NPs. Cells are stained with Phalloidin (actin, green) and DAPI (nuclei, blue). NPs intrinsic emission is visible in red. Focal planes are acquired from the bottom of the cells (upper left, 1) to the top interface with the extracellular bath (lower right, 12). The most part of NPs fluorescence is noticeable in the Z-planes corresponding to the cell inner part (images from 4 to 8), indicating NPs internalization. Scale bars, 10 μm.

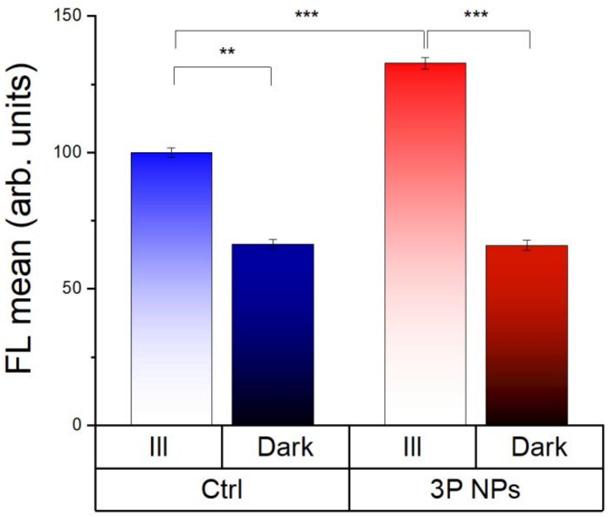

Figure S4. Intracellular ROS production, determined by intracellular APF probe, measured in control untreated cells (Ctrl), and in 3P NPs, in both dark and illuminated condition (|||). Data are represented as mean ± SE values, over statistical samples of n = 56 (control), n = 48 (3P NPs), where n represents the number of cells over three biological replicas for each condition. Statistical significance has been evaluated by one-way ANOVA analysis followed by post-hoc Tukey test. P-values of the test are assigned as follows: ** for $p < 0.02$, *** for $p < 0.001$.

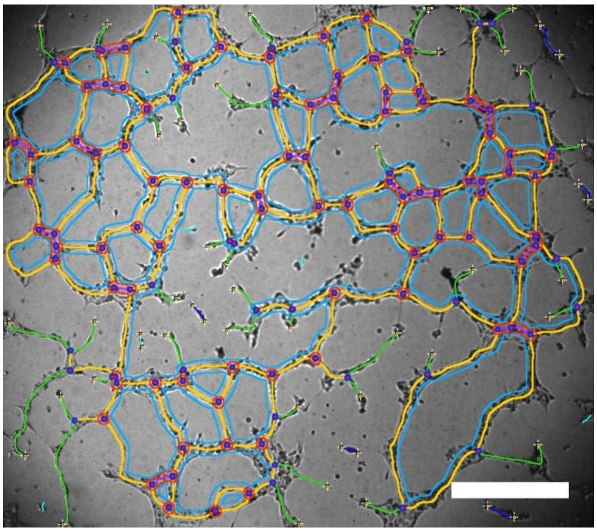

Figure S5. Representative graphical representation of the detected and vectorized components of the HUVECs capillary-like network, superimposed to the correspondent bright field image. Scale bar: 500 μm.
